# Rapid Starch Particle Sizing by YOLOv8n

**DOI:** 10.1016/j.crfs.2026.101491

**Published:** 2026-07-02

**Authors:** Xuyan Zhao, Hanwen Niu, Wenlu Zhu, Chunguang Wang, Xiangyang Li, Kun Xu, Qunfeng Niu, Li Wang, Yuan Zhang

**Affiliations:** a School of Mechanical and Electrical Engineering, Henan University of Technology, Zhengzhou, Henan, 450001, PR China; b College of Electrical Engineering, Henan University of Technology, Zhengzhou, Henan, 450001, PR China; c Henan Women's Vocational College, Zhengzhou, Henan, 450001, PR China

**Keywords:** Starch particle analysis, YOLOv8n, Image segmentation, Attention mechanism, Rapid detection

## Abstract

Accurate measurement of starch particle size is essential for quality control in the food industry. This study developed a rapid image-based detection and analysis method for starch particles based on an improved YOLOv8n network. The model incorporates a P2 small-object detection layer, a simple attention module (SimAM), and the Wise Intersection over Union v3 (WIoU3) loss function to balance segmentation accuracy and computational efficiency. Experimental results showed that the improved model reached an mAP@0.85 of 93.31%, which is 3.50 percentage points higher than that of the baseline, while reducing the number of parameters by about 32.8% and achieving an inference time of 2.4 ms per image on an RTX 3080 GPU. Compared with several lightweight models, the improved model showed higher boundary-sensitive segmentation accuracy while maintaining relatively low model complexity. Comparison with the laser particle size analyzer (LPSA) showed a relative error of 1.85% in the median diameter (D50) and similar particle size distribution trends under the tested conditions. Moreover, the method enabled quantitative analysis of particle circularity, showing a consistent peak near 0.85 across different image-set sizes. Overall, the proposed approach provides a rapid image-based method for starch particle characterization and shows potential for particle-size and morphology analysis in food-related applications.

## Highlights

• Lightweight YOLOv8n enhanced with P2 layer, SimAM, and WIoU3.
• Achieves 93.31% mAP@0.85 with 32.8% fewer parameters and 2.2 ms inference.
• Shows high agreement with BT-9300H laser analyzer (D50 error 1.85%).
• Enables circularity quantification (peak at 0.85) beyond laser techniques.
• Provides an efficient tool for real-time starch particle quality control.

## Graphical abstract

A high-throughput framework for starch particle characterization was established by integrating microscopic imaging and an improved YOLOv8n segmentation model.

**Figure:**
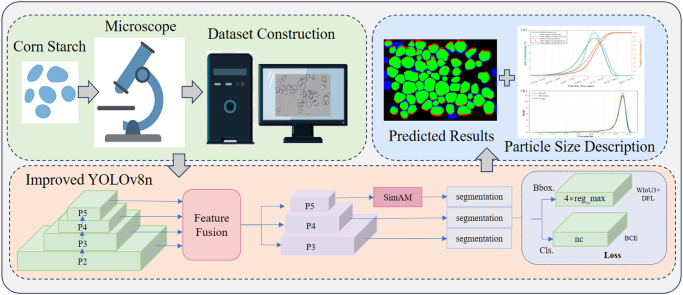

## 1 Introduction

Starch is the primary carbohydrate synthesized by plants via photosynthesis and serves as an important energy reserve widely utilized in the food, feed, and pharmaceutical industries (Bangar et al., 2024). The size and distribution of starch particles are key parameters affecting their functional properties, directly influencing water absorption, gelatinization behavior, and the texture and nutritional quality of final products (Desam et al., 2021; Hu et al., 2025; Ma et al., 2022). Therefore, accurate measurement of starch particle size distribution is essential for improving product quality (Laurène et al., 2025; Protonotariou et al., 2021).

Traditional particle size measurement methods, such as manual sieving or sedimentation methods, are typically labor-intensive and inefficient, making them unsuitable for rapid and high-throughput analysis (Dong et al., 2021; Farooq et al., 2025). Although modern instruments such as laser diffraction and ultrasonic analyzers offer high measurement accuracy, they are costly, require complex sample preparation, and are unable to provide particle morphology information (Pereira and Del Pino Beleia, 2021; Shukla et al., 2010). Consequently, machine vision and deep learning technologies, with their advantages of efficiency, low cost, and automated image interpretation, have emerged as promising approaches for particle size analysis (Shuyuti et al., 2024; Sunil et al., 2024). These technologies have been successfully applied in particle detection across various fields, including materials science, mineral processing, and pharmaceutical production (Ficzere et al., 2023; Li et al., 2022; Neuendorf et al., 2023). Recent studies on image-based particle size detection have also explored multimodal sensing strategies. For instance, Liu et al. (2023) developed an RGB-laser fusion segmentation system with feature dual-recalibration for blast furnace materials, demonstrating the value of multimodal feature fusion for particle boundary perception in complex industrial scenes. However, such RGB-laser systems are mainly designed for macroscopic industrial bulk materials. In contrast, food powder analysis, particularly for micron-scale starch granules, faces distinct challenges associated with weak particle boundaries, adhesion, and morphological variability. Therefore, there remains a need for efficient and microscope-compatible vision solutions that can support particle segmentation and morphological characterization in food applications (Houngbo et al., 2024).

To improve particle size estimation accuracy, various segmentation models have been developed for different application scenarios. Studies have shown that instance segmentation networks such as Mask R-CNN and PANet perform well on complex mineral and synthetic particles (Rosenberger et al., 2024; Zhao et al., 2022), whereas U-Net and its variants (e.g., Res-UNet, TransUNet) are effective for adhesive industrial particles such as coal powder and slag (Fan et al., 2023;Wang et al., 2023). These studies provide important methodological references for addressing challenges such as particle adhesion, overlap, and morphological variation. However, these models generally have complex architectures and a large number of parameters, which may limit their use in rapid image-based particle analysis where computational efficiency and lightweight design are important.

In contrast, YOLOv8 offers notable advantages for rapid particle image analysis due to its fast inference speed and lightweight architecture, as demonstrated in studies on coal and synthetic diamonds (Wu et al., 2024; Zhang et al., 2024). Nevertheless, the original YOLOv8n model still struggles to accurately segment highly adhesive and morphologically varied starch particles, often resulting in missed detections of small targets and blurred boundaries, thereby limiting its application in starch particle size estimation and morphology analysis. Furthermore, most existing studies focus on material or mineral particles, while systematic research on typical food particles such as starch remains limited. Moreover, most of these studies emphasize segmentation accuracy alone, with insufficient validation of the practical reliability of particle size measurements derived from segmentation results.

Based on these considerations, this study developed an improved YOLOv8n framework for rapid starch particle segmentation and particle-size estimation. Microscopic starch images commonly contain small granules, adhesive particle clusters, and weak boundary contrast, which can reduce the accuracy of contour localization. To address these issues, a P2 detection head was used to retain fine spatial information for small particles, SimAM was introduced to enhance particle-related responses and suppress background interference, and WIoU3 was adopted to improve localization stability for particles with different sizes and adhesion states. The proposed method aims to balance segmentation accuracy, model complexity, and processing efficiency, providing an image-based tool for rapid particle-size and morphology analysis in starch and food powder applications.

## 2 Materials and methods

This section describes the complete workflow of the proposed image-based starch particle analysis method, including sample preparation, microscopic image acquisition, image preprocessing, dataset annotation, model construction, training and evaluation settings, and particle-size calculation and validation.

### 2.1 Image acquisition

Commercial corn starch (Xinyang brand, Yonghui Supermarket, Zhengzhou, China) with a particle size range of 5–50 μm was used as the experimental material. A 0.5% (w/w) suspension was prepared by dispersing the starch in deionized water under continuous stirring to achieve a homogeneous mixture. All imaging sessions were performed in a controlled laboratory environment in Zhengzhou, China (ambient temperature ∼20 °C, relative humidity ∼50%) during March. To prevent artifacts from water evaporation, each prepared suspension sample was promptly transferred onto a clean glass slide, formed into a thin layer, and then subjected to microscopic imaging within 30 min.

The imaging system consisted of an NE710 biological microscope (NOVEL Optical Instruments Co., Ltd., Beijing, China) equipped with a 40× Plan Achromat objective (numerical aperture NA = 0.65, field of view approximately 320 × 240 μm) and integrated with an MV-CE100-30 GC industrial camera (Hikvision Digital Technology Co., Ltd., Hangzhou, China) featuring a 10-megapixel sensor and a GigE interface. Image capture and parameter control were performed using MVS software.

**Fig. 1:**
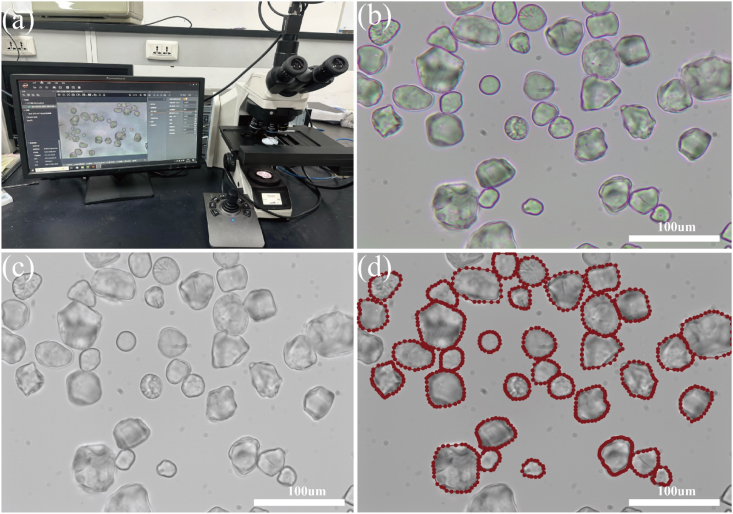
Image acquisition and annotation process. (a) Imaging system based on a microscope and industrial camera. (b) Raw corn starch image. (c) Preprocessed image. (d) Annotated image.

The focus and illumination intensity were adjusted according to sample clarity before image acquisition. The camera exposure time was set to 8 ms after preliminary adjustment and then kept constant across all imaging sessions to maintain consistent acquisition conditions. Gamma correction was applied with a gamma value of 0.8 to enhance contrast in low-intensity boundary regions while avoiding over-saturation of particle interiors. High-resolution images were manually captured and stored for subsequent analysis. Fig. 1(a) illustrates the overall imaging setup, while Fig. 1(b) presents a representative raw image.

### 2.2 Image preprocessing

To address common issues such as shadows, impurities, and uneven illumination in the raw images, a preprocessing pipeline was implemented to enhance segmentation quality. This process included grayscale conversion and bilateral filtering. First, the RGB images were converted to grayscale to reduce redundancy and emphasize particle edges. Bilateral filtering was then applied to suppress noise while effectively preserving boundaries, using a 9-pixel filter diameter and sigma values of 75 for both the intensity and spatial domains, following the OpenCV implementation cv2.bilateralFilter. The resulting preprocessed image is shown in Fig. 1(c). The same preprocessing pipeline was applied to all images during training and inference to maintain input consistency.

### 2.3 Dataset construction and annotation

To support model training for starch particle segmentation, a diverse corn starch image dataset was constructed using the custom-built imaging system. A total of 958 high-resolution images were collected under a range of conditions, including isolated particles, mild adhesion, and severe adhesion. The images were acquired from multiple independently prepared microscope slides. Fields of view with sufficient spatial separation were selected to reduce data correlation caused by adjacent microscopic regions. In total, 22,759 individual particles were manually annotated for training and evaluation, ensuring sufficient variability and complexity. Before model training, 250 images were reserved as an independent test set for final model evaluation and particle size analysis. The remaining 708 images were used to construct the training and validation sets in an approximately 8:2 ratio.

Annotations were performed using the Labelme tool, with particle contours manually traced as polygons by clicking along the boundaries to form closed shapes. Particles intersecting the image border or showing incomplete contours were retained in the raw images, but they were not annotated as target instances and were not included in particle size statistics. Annotation files were saved in JSON format with filenames identical to those of the corresponding images to streamline batch processing. Fig. 1(d) illustrates an annotation example, with red contours delineating particle boundaries and adhesion patterns.

### 2.4 Improved YOLOv8n model

**Fig. 2:**
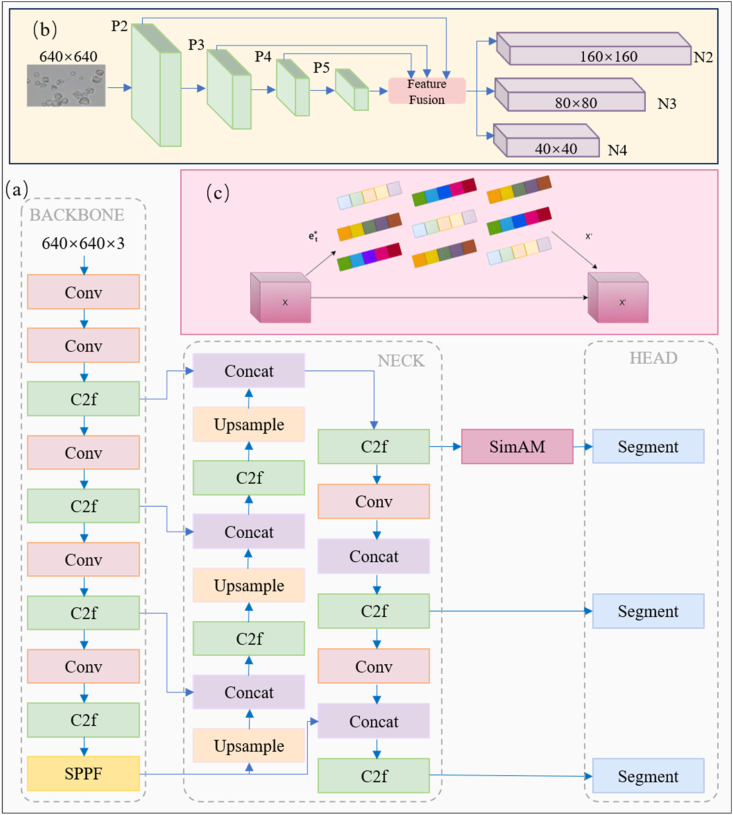
Architecture of the improved YOLOv8n model. (a) Overall network structure. (b) Enhanced detection layer with the P2 small-object module. (c) Structure of the SimAM attention mechanism.

Building upon the baseline YOLOv8n framework, we propose an advanced instance segmentation network specifically tailored for the accurate identification of granular structures. The comprehensive architecture of this improved model is depicted in Fig. 2.

To enhance the adaptability of YOLOv8n for segmenting adhered corn starch particles and improve its boundary localization capability, three key structural modifications were introduced: (1) a P2 small-object detection layer was integrated to capture fine-grained details; (2) the SimAM attention module was incorporated to suppress background noise and improve focus on adhesion regions; and (3) the original complete intersection over union (CIoU) loss was replaced with the WIoU3 loss function to stabilize training and enhance regression accuracy across varying particle sizes. The overall architecture of the proposed model is shown in Fig. 2.

#### 2.4.1 Integration of a P2 small-object detection layer

The original YOLOv8n model performs detection primarily on feature maps at scales P5, P4, and P3, corresponding to spatial sizes of 20 × 20, 40 × 40, and 80 × 80, respectively. However, this configuration is often insufficient for extracting detailed features of small corn starch particles, especially in regions where particles adhere to each other. Such limitations can lead to decreased segmentation accuracy (Jocher et al., 2023a). To address this limitation, a P2 small-object detection layer was introduced into the neck of YOLOv8n. Specifically, the modified detection head replaces the original P5 detection head operating on the 20 × 20 feature map with a P2 detection head operating on the 160 × 160 feature map. This design was used to strengthen small-particle feature localization while avoiding the additional complexity that would be introduced by simply adding an extra detection head. Located at a shallower level, the P2 layer generates high-resolution feature maps that fuse with deeper semantic features, retaining fine-grained edge information while enhancing contextual understanding. This improvement enables more precise boundary localization of small and adhesive particles, leading to better detection and segmentation performance. As shown in Fig. 2(b), the P2 layer generates a 160 × 160 feature map, suitable for detecting objects larger than 4 × 4 pixels. This allows the model to better focus on small targets and improves detection accuracy.

#### 2.4.2 Incorporation of SimAM attention module

SimAM is a lightweight attention mechanism that enhances feature representation by efficiently modeling both channel and spatial information without introducing a heavy parameterized attention branch (Yang et al., 2021). As illustrated in Fig. 2(c), integrating SimAM enables the model to selectively focus on semantically significant regions, which is crucial for accurately segmenting adhered starch particles. Unlike conventional attention modules (e.g., SE, CBAM), SimAM assesses the importance of each neuron directly through a computationally efficient energy function. This function quantifies the linear separability between a target neuron and its neighbors within each channel, assigning higher weights to neurons that are more discriminative.

For an input feature map, the energy $e_{t}$ for a target neuron t is defined as:

$$e_{t}=\frac{4\left({\hat{\sigma }}^{2}+\lambda \right)}{{\left(t-\hat{\mu }\right)}^{2}+2{\hat{\sigma }}^{2}+2\lambda }$$

where $\hat{\mu }$ and ${\hat{\sigma }}^{2}$ represent the mean and variance of all neurons in the same channel as t, calculated as:

$$\hat{\mu }=\frac{1}{n}\sum_{i=1}^{n}\;x_{i},{\hat{\sigma }}^{2}=\frac{1}{n}\sum_{i=1}^{n}\;{\left(x_{i}-\hat{\mu }\right)}^{2}$$

In these equations, n denotes the number of neurons in the channel, $x_{i}$ represents other neurons within the same channel, and λ is a regularization hyperparameter.

A lower energy value $e_{t}$ indicates higher importance of the target neuron. The final output feature map is obtained by applying a sigmoid function to the normalized energy values:

$$\tilde{X}=\sigma \left(\frac{1}{E}\right)⊙X$$

where E contains all energy values $e_{t}$ in the feature map, σ is the sigmoid function, and ⊙ denotes element-wise multiplication.

By suppressing less informative background features and emphasizing those that correspond to particle boundaries and key structures, the module significantly improves the network's ability to resolve overlapping regions and to refine boundary localization.

#### 2.4.3 Adoption of the WIoU loss function

The original YOLOv8n model utilizes the CIoU loss, which extends the basic Intersection over Union (IoU) metric by incorporating geometric factors such as the distance between the center points and the consistency of aspect ratios. While effective in many scenarios, CIoU exhibits limitations when dealing with starch particles. Its reliance on aspect ratio can become uninformative when the predicted and ground-truth boxes share similar proportions, thereby limiting its capacity to guide precise bounding box adjustments. More critically, in cases of low initial IoU, which are common with small or highly overlapping particles, the aspect ratio term can disproportionately dominate the gradient, potentially leading to unstable training and suboptimal convergence.

To overcome these drawbacks, we adopted the WIoU loss function (Tong et al., 2023). The WIoU loss function is formulated as:

$$\begin{array}{c} L_{WIoU}=R_{WIoU}L_{IoU}\\ R_{WIoU}=\exp\left(\frac{{\left(x-x_{gt}\right)}^{2}+{\left(y-y_{gt}\right)}^{2}}{{\left(W_{g}^{*}\right)}^{2}+{\left(H_{g}^{*}\right)}^{2}}\right)\\ L_{IoU}=1-IoU\\ IoU=\frac{\left|B\cap B^{gt}\right|}{\left|B\cup B^{gt}\right|} \end{array}$$

where $R_{\text{WIoU}\;}$ is the penalty term, $L_{IoU}$ is the primary loU loss, (x,y) and ($x_{gt},y_{gt}$) are the center coordinates of the predicted box B and the ground-truth box $B^{gt}$, respectively, $W_{g}$ and $H_{g}$ are the dimensions of the smallest enclosing box covering both B and $B^{gt}$, the * symbol denotes a detach operation preventing gradient assignment to enclosing box size.

WIoU introduces a dynamic focusing mechanism that assigns adaptive gradient gains according to sample localization quality. This mechanism reduces the contribution of well-localized samples that provide limited additional learning information, while increasing the optimization weight of lower-quality samples, such as small, partially occluded, or adhered particles. By rebalancing the learning focus in this manner, WIoU can improve regression stability and support more accurate localization of particle regions, which is important for subsequent size and shape analysis of adhered corn starch particles.

### 2.5 Experimental setup

The experiments in this study were conducted on a Windows 10 platform, with PyTorch serving as the primary framework for model training and evaluation. The system was configured with an Intel i7-12700K processor, an NVIDIA GeForce RTX 3080 GPU with 10 GB of video memory, and 128 GB of system RAM. The software environment included PyCharm IDE, Python 3.8, and PyTorch version 1.10.0.

To improve the model's generalization capability and robustness, the order of training images was randomized to prevent sequential biases during learning. The input image resolution was set to 640 × 640 pixels. The training was performed over 100 epochs with a batch size of 16. The learning rate was initialized at 0.01, and the momentum parameter was set to 0.937. The number of data loading threads was set to 0. The Adam optimizer was employed to speed up convergence by incorporating momentum from past gradients. After each training epoch, model performance was assessed on the validation set, and both the final epoch weights and the best-performing weights were saved.

### 2.6 Evaluation metrics

Model performance was comprehensively evaluated using precision, recall, F1-score, mask mean average precision (mask mAP) at IoU thresholds of 0.5 and 0.85 and averaged over 0.5:0.95, along with model complexity and efficiency metrics, including parameters, FLOPs, GPU memory usage, and inference speed. The reported mAP values refer to mask mAP calculated from segmentation masks rather than box mAP calculated from bounding boxes. The stringent mAP@0.85 was prioritized as the key metric to evaluate boundary segmentation fidelity for starch particles, while precision, recall, and F1-score provided additional insights into instance-level segmentation performance. The main formulations are given below.

Precision was calculated as:

$$\text{Precision}=\frac{\text{TP}}{\text{TP}+\text{FP}}$$

where TP and FP denote true positives and false positives, respectively.

Recall was calculated as:

$$\text{Recall}=\frac{\text{TP}}{\text{TP}+\text{FN}}$$

where FN denotes false negatives.

F1-score was calculated as:

$$F1=\frac{2\times \text{Precision}\times \text{Recall}}{\text{Precision}+\text{Recall}}$$

Mean average precision at a given IoU threshold θ was calculated as:

$${\text{mAP}}_{\theta }=\frac{1}{C}\sum_{c=1}^{C}{\text{AP}}_{c}\left(\theta \right)$$

where C is the number of classes and ${\text{AP}}_{c}\left(\theta \right)$ is the average precision of class c at IoU threshold θ. In this study, the target class was starch particles.

To more directly evaluate segmentation quality in the context of particle-size measurement, four additional particle-level metrics were adopted: Mask IoU (M-IoU), Boundary F-score (BF), Hausdorff distance (HD), and equivalent diameter error (EDE).

Mask IoU measures the pixel-level overlap between predicted and ground-truth masks averaged over all matched particle instances:

$$M‐\text{IoU}=\frac{1}{K}\sum_{k=1}^{K}\frac{\left|{\hat{M}}_{k}\cap M_{k}\right|}{\left|{\hat{M}}_{k}\cup M_{k}\right|}$$

where ${\hat{M}}_{k}$ and $M_{k}$ denote the predicted and ground-truth masks of the k-th matched particle instance, respectively, and K is the number of matched particle instances.

BF was used to evaluate boundary accuracy by calculating the F-score of predicted contour points within a tolerance distance of 2 pixels from the ground-truth boundary. HD was used to quantify the maximum boundary deviation between the predicted and ground-truth contours for each particle instance. EDE was used to quantify particle-size-related segmentation error based on equivalent diameter. These particle-level metrics provide complementary evaluation of mask overlap, boundary localization, contour deviation, and particle-size-related segmentation accuracy. The detailed formulations of BF, HD, and EDE are provided in Appendix A.1.

### 2.7 Comparative experiments and ablation study

To systematically validate the design choices and overall effectiveness of the proposed model, a series of experiments were conducted under identical hardware and hyperparameter settings, including the same dataset, input resolution, learning rate, and batch size.

#### 2.7.1 Comparison of attention mechanisms

To investigate the impact of different attention mechanisms on feature extraction and target localization, four lightweight attention modules were embedded near the N2 detection head in the YOLOv8n backbone. These modules included the Self-Attention (SA) module, Convolutional Block Attention Module (CBAM), Global Attention Module (GAM), and SimAM. These modules were tested to enhance spatial and channel-wise focus while preserving critical feature information. By comparing segmentation performance and boundary localization accuracy across these attention variants, this experiment evaluates the suitability and advantages of SimAM for particle-level segmentation and supports the selection of an optimal bounding-box refinement strategy.

#### 2.7.2 Comparison of loss functions

To further improve the robustness and accuracy of bounding-box regression in complex particle distributions, especially for small or overlapping particles, the original CIoU loss function was replaced with WIoU. Three WIoU variants were evaluated. WIoU1 applies a basic gradient-weighting scheme for generally separated samples. WIoU2 introduces dynamic weighting for targets with extreme aspect ratios or large scale variance. WIoU3 refines the dynamic weighting to emphasize small-object detection and to better handle occluded or adherent particles. These experiments aimed to identify the most effective weighting strategy for bounding-box optimization in the present particle segmentation task.

#### 2.7.3 Ablation study on integrated components

An ablation study was designed to evaluate the individual and combined contributions of the three proposed components, including the P2 detection head, SimAM attention module, and WIoU3 loss function. Eight model configurations were evaluated, including the baseline YOLOv8n, three single-module variants, three pairwise-module variants, and the final integrated model. All configurations were trained and evaluated under the same dataset split, input resolution, and hyperparameter settings to ensure a fair comparison of segmentation accuracy, model efficiency, and particle-level segmentation quality.

#### 2.7.4 Performance comparison with different segmentation algorithms

To place the final model in the context of existing solutions, it was compared with several mainstream lightweight segmentation networks, including YOLOv5n (Jocher, 2020), YOLOv7n (Wang et al., 2023), YOLOv8s, and YOLOv11n (Jocher et al., 2023b). This comparative analysis evaluated the relative performance and competitiveness of the proposed improved YOLOv8n model for corn starch particle segmentation.

### 2.8 Particle size measurement and validation

To ensure accurate dimensional analysis of starch particles, spatial calibration was performed because pixel values do not directly represent real-world dimensions. A 100 μm μm scale was included in the imaging field, from which a pixel-to-micrometer conversion factor was obtained (2393 pixels = 100 μm, corresponding to 0.0418 μm/pixel, approximately 0.04 μm/pixel).

After segmentation, geometric parameters were extracted to characterize particle size and morphology. Common descriptors include particle diameter, median diameter (D50), and size distribution, as well as shape factors such as circularity (C. Wang et al., 2023; J. Wang et al., 2019). The main geometric parameters were calculated from the predicted particle masks after spatial calibration. Particle area was calculated as:

$$S=n\times d^{2}$$

where n is the number of pixels in the particle mask and d is the calibration factor (μm/pixel). Circularity was calculated as:

$$R=\frac{\sqrt{4S\pi }}{P}$$

where *P* is the particle perimeter. Equivalent diameter was calculated as:

$$D_{eq}=\sqrt{\frac{4S}{\pi }}$$

The particle size distribution was calculated as:

$$f\left(D\right)=\frac{N_{D}}{N_{\text{total}}}$$

where $N_{D}$ is the number of particles in size interval D, and $N_{\text{total}}$ is the total number of particles in the sample. The median diameter D50 was defined as the particle diameter corresponding to the 50th percentile of the cumulative size distribution.

A total of 250 images were used for particle-size measurement and validation. These images corresponded to the independent test set reserved before model training, as described in Section 2.3, and were not used for training, validation, or model selection. The proposed segmentation model identified individual particles, and their geometric features were extracted using OpenCV. Pixel-based measurements were converted to real-world dimensions using the calibration factor.

To validate the accuracy of the proposed method, a BT-9300H laser particle size analyzer (LPSA) was employed as the reference instrument. For the image-based method, five groups of images (50 per group, ∼1000 particles each) were randomly selected from the dataset and analyzed independently. The laser analyzer measured the same sample five times, and the results were averaged for comparison.

This setup allowed for a direct evaluation of:

**Key size metrics**: Including the D50, area-based mean diameter, and length-based mean diameter.

**Comparison of particle size distribution curves:** For the particle size distribution analysis, a representative laser reference was established by averaging the five distribution curves obtained from repeated laser measurements. Concurrently, the proposed image-based method generated its distribution profile by extracting and fitting particle data from the entire set of 250 images. This allowed for a direct comparison of the overall distribution characteristics between the two methods.

**Morphological analysis**: The proposed method was further utilized to evaluate particle circularity, a morphological attribute inaccessible to laser diffraction. To assess the stability of circularity measurements, analyses were conducted across three datasets of varying scales: small (50 images), medium (150 images), and large (250 images). This multi-scale approach ensured the reliability and sample-size robustness of the shape characterization.

## 3 Results and discussion

This section presents the experimental results and discussion in sequence, including the effects of the attention mechanism and loss function, the ablation study, comparison with other segmentation models, starch particle-size measurement, particle-size distribution validation, and circularity analysis.

### 3.1 Effect of attention mechanisms

To evaluate the impact of different attention mechanisms on starch particle segmentation performance, comparative experiments were conducted as summarized in Table 1. Among the tested attention modules, SimAM achieved the best overall balance between segmentation accuracy and model complexity, with the highest recall (97.82%), F1-score (97.33%), and mAP@0.85 (90.94%). Compared with the baseline, SimAM improved mAP@0.85 by 1.13 percentage points while reducing the profiled number of model parameters by 5552. Although other attention modules showed marginal gains in certain metrics, their overall performance was less balanced than that of SimAM.

**Table 1:** Comparison of experimental results using different attention mechanisms.

Models	Precision (%)	Recall (%)	F1-score (%)	mAP@0.5 (%)	mAP@0.85 (%)	mAP@0.5:0.95 (%)	Parameters
base	96.52	97.69	97.10	98.86	89.81	85.76	3136771
base-SA	96.45	97.70	97.07	98.91	89.96	85.75	3136795
base-CBAM	96.77	97.29	97.03	98.77	90.29	85.80	3137450
base-GAM	96.93	97.18	97.05	98.73	90.22	85.72	3164227
**base-SimAM**	**96.85**	**97.82**	**97.33**	**98.90**	**90.94**	**85.86**	**3131219**

**Fig. 3:**
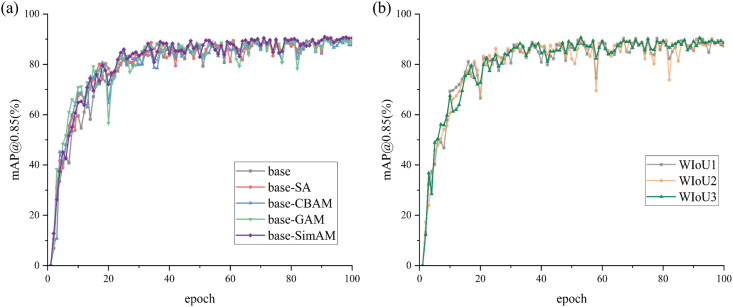
Performance comparison under different configurations. (a) mAP@0.85 curves with various attention mechanisms. (b) mAP@0.85 curves with different WIoU variants.

The advantage of SimAM can be attributed to its energy function design. This module estimates neuron importance based on linear separability, enabling joint spatial and channel attention without introducing a heavy parameterized attention branch. When processing adhesive starch particles, the mechanism enhances particle-related responses and suppresses background interference, thereby improving boundary-sensitive segmentation performance. The training curve in Fig. 3(a) further shows that SimAM maintained a relatively high mAP@0.85 during the later training stages, indicating stable convergence under the tested training setting.

### 3.2 Performance of WIoU loss function

To investigate the effect of different loss functions on the optimization of boundary regression for adhesive corn starch particles, three variants of the WIoU loss were compared under a unified model framework. To examine the influence of random initialization, the key boundary-sensitive metric mAP@0.85 was further evaluated using five random seeds.

As shown in Table 2, WIoU3 achieved the best overall performance, with an mAP@0.85 of 90.55%, representing improvements of 0.16% and 0.32% over WIoU1 and WIoU2, respectively. It also demonstrated more stable regression behavior while maintaining high accuracy, as illustrated in Fig. 3(b). The repeated experiments further showed that WIoU3 achieved the highest mean mAP@0.85 (90.55 ± 0.10%), compared with WIoU1 (90.39 ± 0.15%) and WIoU2 (90.23 ± 0.13%). Although the improvement over WIoU1 was relatively small, WIoU3 showed more stable boundary-sensitive performance across the repeated runs.

**Table 2:** Performance comparison of different WIoU variants.

Loss	Precision (%)	Recall (%)	F1-score (%)	mAP@0.5 (%)	mAP@0.85 (%)	mAP@0.5:0.95 (%)
WIoU1	96.11	97.31	96.70	98.72	90.39	85.67
WIoU2	96.41	97.47	96.93	98.80	90.23	85.89
**WIoU3**	**96.74**	**97.89**	**97.31**	**98.86**	**90.55**	**86.04**

The performance advantage of WIoU3 may be attributed to its dynamic focusing mechanism, which adjusts gradient gains according to sample localization quality. This design reduces the influence of ordinary-quality samples during loss optimization and allows the model to focus more effectively on difficult cases, such as small particles and particles with severe adhesion. In contrast, WIoU1 lacks dynamic adjustment capability, while WIoU2 uses a monotonic focusing strategy that may not sufficiently distinguish samples with different localization quality, resulting in limited improvement in adhesive boundary segmentation.

### 3.3 Ablation study on integrated components

Based on the ablation design described in Section 2.7.3, the contribution of each improvement module was evaluated on the corn starch particle dataset. The results are summarized in Table 3, Table 4. Table 3 reports the segmentation accuracy metrics, while Table 4 reports model efficiency and particle-level segmentation metrics.

**Table 3:** Ablation results of model configurations.

Model	YOLOv8n	P2	SimAM	WIoU3	Precision (%)	Recall (%)	F1-score (%)	mAP@0.5(%)	mAP@0.85(%)	mAP@0.5:0.95(%)	Parameters
1	✓				96.52	97.69	97.10	98.86	89.81	85.76	3136771
2	✓	✓			96.72	97.04	96.88	98.88	92.22	86.37	1813814
3	✓		✓		96.45	97.82	97.13	98.89	90.94	85.86	3131219
4	✓			✓	96.74	97.89	97.31	98.86	90.55	86.04	3136771
**5**	✓	✓	✓		96.81	97.61	97.21	98.90	93.00	87.14	1808262
**6**	✓	✓		✓	96.83	97.35	97.09	98.89	92.88	86.92	1813814
**7**	✓		✓	✓	96.68	97.91	97.29	98.90	91.60	86.47	3131219
**8**	**✓**	**✓**	**✓**	**✓**	**96.89**	**97.72**	**97.30**	**98.91**	**93.31**	**87.92**	**2107043**

**Table 4:** Efficiency and particle-level metrics of model configurations.

Model	YOLOv8n	P2	SimAM	WIoU3	FLOPs (G)	GPU Mem. (MB)	FPS	M-IoU (%)	BF (%)	HD (px)	EDE (μm)
1	✓				12.4	1847	400	88.74	85.32	4.87	0.94
2	✓	✓			9.8	1624	431	90.21	87.15	4.23	0.81
3	✓		✓		12.4	1843	399	89.56	86.44	4.51	0.88
4	✓			✓	12.4	1847	400	89.18	86.89	4.39	0.85
**5**	✓	✓	✓		9.7	1619	434	90.84	88.02	4.01	0.76
**6**	✓	✓		✓	9.8	1624	431	90.63	87.94	4.08	0.78
**7**	✓		✓	✓	12.4	1843	399	90.12	87.61	4.27	0.83
**8**	**✓**	**✓**	**✓**	**✓**	**10.1**	**1698**	**421**	**91.63**	**89.27**	**3.74**	**0.72**

Overall, mAP@0.5 was already close to saturation for all models, whereas mAP@0.85 and mAP@0.5:0.95 provided more discriminative evidence for boundary-sensitive segmentation performance. Among the single-module variants, P2 replacement showed the largest increase in mAP@0.85, improving it from 89.81% to 92.22%. This improvement may be attributed to the use of a higher-resolution detection head for small particle localization. Meanwhile, the parameter count was reduced because the original P5 detection head was replaced rather than retained. Although the P2 head operates on a higher-resolution feature map, it uses lower-channel shallow features, whereas the removed P5 head is connected to deeper high-channel features and contains heavier convolutional layers. Therefore, replacing P5 with P2 resulted in a net reduction of 1,322,957 trainable parameters in Model 2 compared with the baseline. SimAM and WIoU3 also improved mAP@0.85, suggesting that feature reweighting and regression loss optimization contributed to boundary-sensitive segmentation.

The pairwise combination results further suggest complementary effects among the three modules. Compared with their corresponding single-module variants, P2+SimAM, P2+WIoU3, and SimAM + WIoU3 all achieved higher mAP@0.85. The final integrated model achieved the highest mAP@0.85 of 93.31% and the highest mAP@0.5:0.95 of 87.92%, indicating that the three components jointly improved segmentation accuracy. To further examine the robustness of the ablation results, the key metric mAP@0.85 was evaluated over five random seeds. The proposed model achieved the highest mean mAP@0.85 (93.31 ± 0.16%), outperforming the baseline (89.81 ± 0.16%), P2 replacement (92.22 ± 0.12%), SimAM (90.94 ± 0.13%), and WIoU3 (90.55 ± 0.15%). These results indicate that the integrated model provided consistently higher boundary-sensitive segmentation performance than the baseline and individual modules under the tested conditions. Its profiled parameter count was 2,107,043, which remained 32.83% lower than that of the baseline YOLOv8n. Overall, the proposed model improved boundary-sensitive segmentation performance while maintaining lower model complexity than the original YOLOv8n.

The efficiency and particle-level metrics in Table 4 further support these observations. P2-based variants reduced FLOPs and GPU memory usage while improving throughput, indicating that replacing the heavier P5 head with a lightweight P2 head decreased the overall computational burden despite the higher spatial resolution of the P2 feature map. The final model achieved the highest Mask IoU and Boundary F-score and the lowest Hausdorff distance and EDE among the tested configurations. These results suggest that the improvements in mAP were also reflected in more accurate particle contour delineation and particle-size-related segmentation quality.

**Fig. 4:**
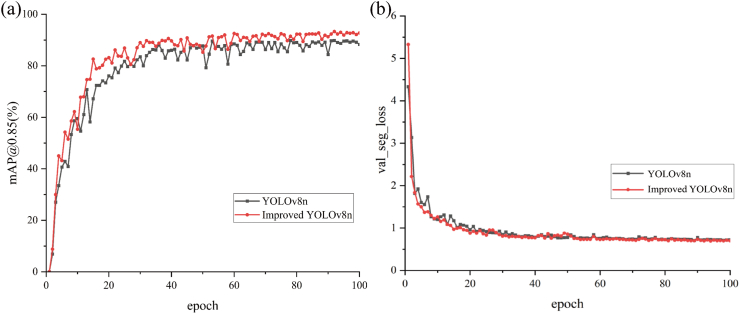
Comparison between baseline YOLOv8n and the improved model. (a) mAP@0.85 comparison. (b) Validation segmentation loss comparison.

To provide a more intuitive comparison, Fig. 4 presents the training performance of the baseline and improved models. As shown in Fig. 4(a), both models converged rapidly, while the improved model maintained higher mAP@0.85 during training. In addition, its validation loss remained lower than that of the baseline model, as shown in Fig. 4(b), suggesting improved validation performance.

**Fig. 5:**
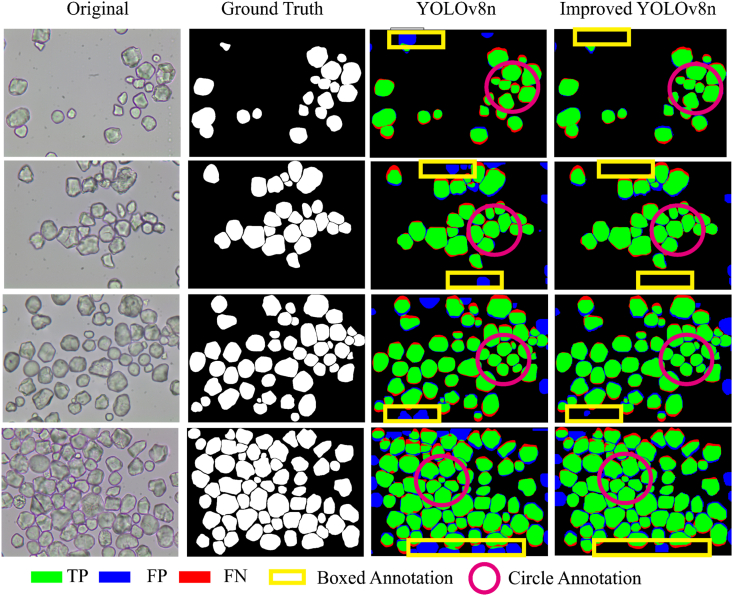
Visualization of starch particle segmentation results obtained by the baseline and improved YOLOv8n models.

Fig. 5 presents a comparative analysis of segmentation performance using four representative images with increasing complexity from Image 1 to Image 4. Green, blue, and red regions correspond to true positives, false positives, and false negatives, respectively. In boundary-incomplete regions, some blue areas arise from predictions on unannotated incomplete boundary particles, which are counted as false positives because no matched ground-truth instances are available. Compared with the baseline model, the improved model reduced these unmatched predictions through enhanced feature representation. For adhesion regions, the improved model achieved more precise boundary localization with fewer false negatives and false positives, which may be attributed to its improved feature extraction and boundary regression capability.

**Fig. 6:**
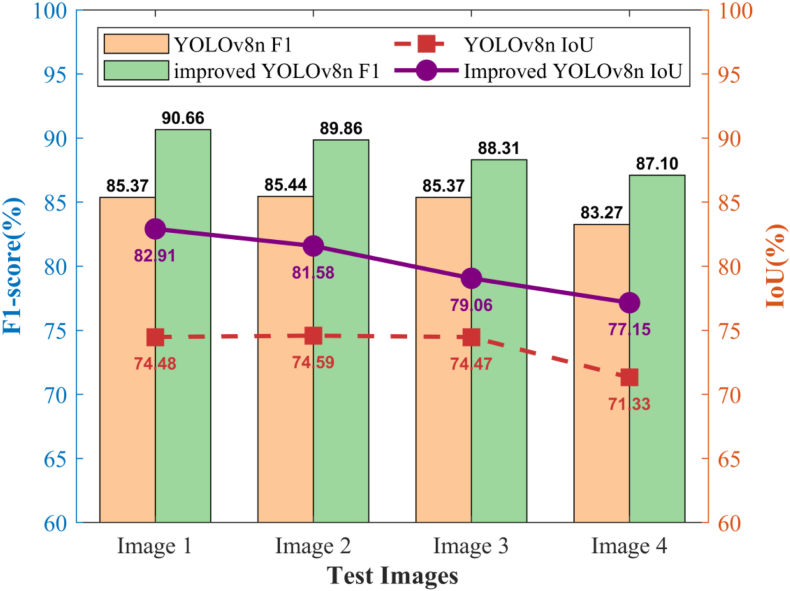
Quantitative evaluation of the segmentation results.

Quantitative results in Fig. 6 further support these observations. The improved model achieved higher F1-score and IoU values than the baseline across all representative test cases, with F1-score improvements of 4.3%–6.2% and IoU improvements of 8.4%–11.4%. Although both models exhibited performance degradation with increasing image complexity, the improved model maintained higher segmentation accuracy in the most challenging case, indicating better performance in handling complex particle adhesion and boundary ambiguity.

### 3.4 Comparison with other segmentation models

The improved YOLOv8n model was compared with several mainstream lightweight segmentation networks to evaluate its performance in corn starch particle analysis. As shown in Table 5, the proposed model achieved higher boundary-sensitive segmentation accuracy than the compared models while maintaining relatively low model complexity.

**Table 5:** Comparison of segmentation performance among different algorithms.

Models	Precision (%)	Recall (%)	F1-score (%)	mAP@0.5(%)	mAP@0.85(%)	mAP@0.5:0.95(%)	Parameters	Time/ms
YOLOv5n	97.23	95.79	96.50	98.66	77.76	80.25	1879750	3.4
YOLOv7n	95.85	96.57	96.21	98.62	77.72	80.23	37842476	5.8
YOLOv8n	96.52	97.69	97.10	98.86	89.81	85.76	3136771	2.5
YOLOv8s	96.76	97.77	97.26	98.86	92.14	86.77	11552595	2.5
YOLOv11n	95.27	96.72	95.99	98.26	92.21	87.25	2834763	2.0
**Improved YOLOv8n**	**96.89**	**97.72**	**97.30**	**98.91**	**93.31**	**87.92**	**2107043**	**2.4**

In terms of segmentation accuracy, the improved YOLOv8n achieved an mAP@0.85 of 93.31%, which was higher than YOLOv5n, YOLOv7n, the original YOLOv8n, YOLOv8s, and YOLOv11n by 15.55, 15.59, 3.50, 1.17, and 1.10 percentage points, respectively. It also achieved the highest mAP@0.5:0.95 among the compared models, indicating good segmentation performance across different IoU thresholds. In terms of model efficiency, the improved YOLOv8n contained 2.11 M parameters, representing a 32.8% reduction compared with the original YOLOv8n. Its parameter count was also lower than those of YOLOv11n (2.83 M), YOLOv7n (37.84 M), and YOLOv8s (11.55 M). The inference time of the proposed model was 2.4 ms per image on the RTX 3080 workstation, which was slightly slower than YOLOv11n (2.0 ms) but comparable to the original YOLOv8n and YOLOv8s.

Overall, the improved YOLOv8n showed a favorable balance between segmentation accuracy, model complexity, and inference speed for corn starch particle analysis. Although YOLOv11n achieved a shorter inference time, the proposed model provided higher mAP@0.85 and mAP@0.5:0.95 with a lower parameter count, supporting its use as an efficient image-based particle segmentation model.

### 3.5 Starch particle size measurement

**Fig. 7:**
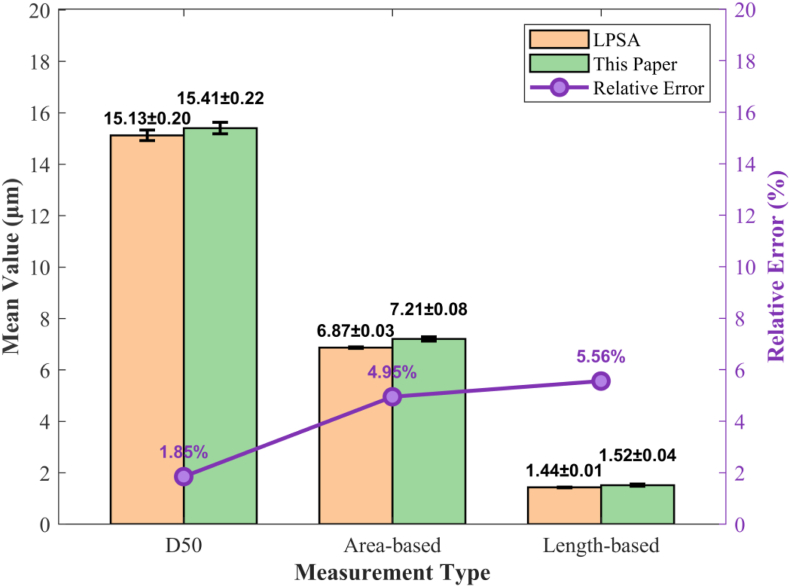
Comparison of particle size indices between the proposed method and the LPSA.

A comparative evaluation between the improved YOLOv8n algorithm and the LPSA showed reasonable agreement across all particle size parameters (Fig. 7). The mean values and standard deviations of D50, area-based mean diameter, and length-based mean diameter obtained by the proposed algorithm showed reasonable agreement with the laser analyzer results. For the key metric of D50, the mean values from both methods were nearly identical, with a relative error of 1.85%. Similarly, the area-based and length-based mean diameters exhibited relative errors of 4.95% and 5.56%, respectively, all remaining below 6%. The small standard deviations observed in repeated measurements suggested acceptable repeatability of the proposed image-based measurement under the tested conditions.

The slight positive deviation observed in the image-based results may be associated with differences in particle boundary definition and measurement principle between the two methods. In this study, to preserve the projected morphological characteristics of starch particles, all annotations were delineated along the visible outer optical contour of each particle (Fig. 1(d)), following the principle of image-based particle analysis (ISO, 2014). This annotation strategy captures the complete projected boundary of the particle, but it may also include weak gray-level transition regions caused by refraction, diffraction, and local edge gradients. In contrast, LPSA estimates equivalent particle size from light-scattering signals using a different measurement principle (ISO, 2020). Therefore, the small difference between the two methods may mainly reflect method-specific differences in boundary definition, projected morphology, and measurement principle.

### 3.6 Validation of particle size distribution

**Fig. 8:**
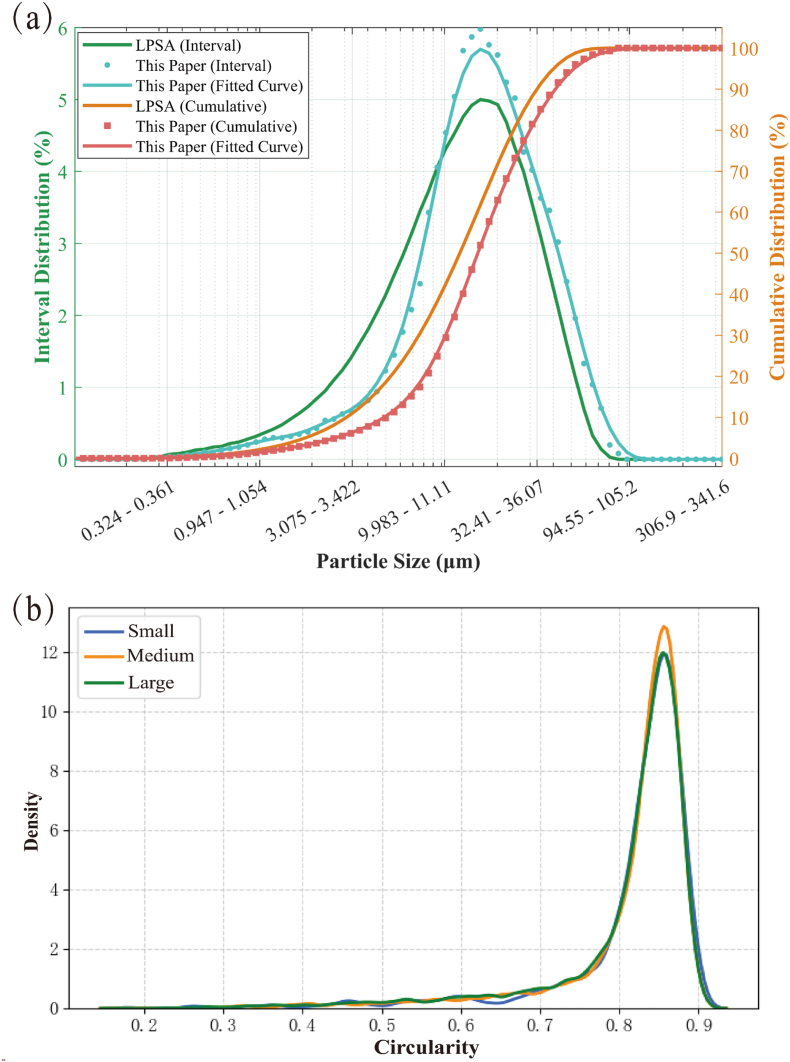
Comparison of the proposed method and the LPSA. (a) Particle size distribution. (b) Circularity distribution across different scales.

To further assess the consistency between the proposed method and the LPSA, Fig. 8(a) compares the cumulative and interval particle size distribution curves obtained by both methods. As shown, the two methods exhibit similar particle size distribution trends. The probability distribution curves reach their peaks within the range of 15 to 18 μm, and the cumulative distribution curves followed similar patterns. This trend-level agreement suggests that the proposed method can provide rapid image-based particle size estimation under the tested conditions.

Closer examination reveals two subtle differences. First, the interval distribution curve obtained by the proposed method shows a slight shift toward larger particle diameters compared with the laser reference. This observation is consistent with the minor positive deviation of the mean diameter values discussed in Section 3.5 and may be associated with the boundary definition and projected morphology captured by the image-based method. Second, the interval distribution generated by the proposed method presents a sharper peak than that of the laser analyzer, indicating a more concentrated particle size distribution. This distinction may be related to the different measurement mechanisms and data acquisition principles of the two methods. LPSA estimates equivalent particle size from light-scattering signals, whereas the proposed image-based method extracts particle size descriptors from individually segmented two-dimensional masks. Therefore, the distribution comparison should be interpreted as a consistency assessment between two different measurement approaches, rather than as a fully equivalent distribution validation.

Overall, the proposed method produced particle size estimates that were generally consistent with the LPSA results under the tested conditions. Together with its high detection efficiency and ability to provide particle-level morphology information, the method shows potential for rapid image-based particle size analysis in food-related applications.

### 3.7 Analysis of particle circularity

The proposed image analysis method not only provides particle size estimates but also enables quantitative assessment of particle circularity, which conventional laser diffraction cannot provide. This additional capability complements traditional size-based measurements and allows complementary characterization of starch particle morphology.

As shown in Fig. 8(b), the circularity values of corn starch particles are mainly concentrated within 0.8 to 0.9, with a distinct peak around 0.85. This distribution pattern remains consistent across small-scale (50 images), medium-scale (150 images), and large-scale (250 images) datasets, confirming the stability and repeatability of the circularity measurement.

Circularity, as a key descriptor of shape regularity, provides new insights into the processing behavior and functional characteristics of starch particles (Argamosa et al., 2023). Nearly spherical particles (circularity close to 1) generally exhibit better flowability and dispersibility, while irregular shapes may affect texture and solubility (Bhat and Riar, 2016). The established circularity distribution thus offers a valuable reference for future studies on the relationship between starch morphology and functional properties. By integrating shape parameters with size analysis, the proposed method enables complementary characterization of starch particles and shows potential for quality control, process optimization, and product development in food and pharmaceutical applications.

## 4 Conclusion

This study developed a rapid detection and analysis method for starch particles based on an improved YOLOv8n architecture. By integrating the P2 small-object detection layer, the SimAM attention mechanism, and the WIoU3 loss function, the proposed model achieved a marked improvement in segmentation accuracy. Its mAP@0.85 reached 93.31%, representing a 3.50% increase over the baseline model, while reducing the number of parameters by approximately 32.8%, FLOPs by 18.5%, and GPU memory by 8.1%, with throughput improved to 421 FPS and an inference time of 2.4 ms per image on an RTX 3080 GPU, showing a favorable balance between accuracy and efficiency. In the validation of particle size measurements, the proposed method showed reasonable agreement with the LPSA under the tested conditions in both D50 and particle size distribution curves. The relative errors of all metrics were below 6%, supporting the feasibility of the measurement results. In addition to particle-size information, the proposed image-based method further quantified morphological descriptors such as particle circularity, which are generally unavailable from laser diffraction measurements, thereby providing complementary information for starch granule characterization.

Overall, this study provides an image-based analysis approach for corn starch particle characterization under the investigated experimental conditions. The proposed method shows potential for starch quality monitoring and particle-size analysis. However, its applicability to other starch varieties, food powders, and imaging systems requires further validation using more diverse datasets and acquisition conditions. Future studies will investigate advanced imaging strategies, including 3D morphological reconstruction, to improve the analysis of complex particle stacking, and will conduct end-to-end latency profiling and edge-device validation to further assess the practical applicability of the proposed method.

## Data availability statement

The dataset annotations, model configuration files, training scripts, and trained weights are available from the corresponding author upon reasonable request, subject to institutional data management requirements.

## Credit author statement

**Xuyan Zhao**: Formal analysis, Writing - original draft, Writing - review & editing.

**Hanwen Niu:** Methodology, Data curation, Validation. **Wenlu Zhu**: Conceptualization, Investigation. **Chunguang Wang:** Visualization. **Xiangyang Li**: Investigation. **Kun Xu**: Formal analysis. **Qunfeng Niu:** Resources, Funding acquisition. **Li Wang:** Funding acquisition, Writing - review & editing. **Yuan Zhang**: Supervision, Project administration.

## Declaration of competing interest

The authors declare that they have no known competing financial interests or personal relationships that could have appeared to influence the work reported in this paper.

## Appendix A

### A.1 Formulations of particle-level segmentation metrics

Boundary F-score evaluates the precision and recall of predicted contour points within a tolerance distance threshold δ, which was set to 2 pixels in this study. Boundary precision $P_{b}$, boundary recall $R_{b}$, and BF were calculated as follows:

$$P_{b}=\frac{\left|\left\{p\in \partial \hat{M}:\min_{q\in \partial M}∥p-q∥\leq \delta \right\}\right|}{\left|\partial \hat{M}\right|}$$

$$R_{b}=\frac{\left|\left\{q\in \partial M:\min_{p\in \partial \hat{M}}∥q-p∥\leq \delta \right\}\right|}{\left|\partial M\right|}$$

$$\text{BF}=\frac{2P_{b}R_{b}}{P_{b}+R_{b}}$$

where $\partial \hat{M}$ and ∂M represent the predicted and ground-truth mask boundary point sets, respectively.

Hausdorff distance measures the maximum boundary deviation between the predicted contour and the ground-truth contour. For the k-th particle instance, HD was calculated as:

$${\text{HD}}_{k}=\max\left\{\max_{p\in \partial {\hat{M}}_{k}}\min_{q\in \partial M_{k}}∥p-q∥,\max_{q\in \partial M_{k}}\min_{p\in \partial {\hat{M}}_{k}}∥q-p∥\right\}$$

The mean HD over all matched particle instances was calculated as:

$$\text{HD}=\frac{1}{K}\sum_{k=1}^{K}{HD}_{k}$$

EDE was used to quantify particle-size-related segmentation error based on equivalent diameter:

$$\text{EDE}=\frac{1}{K}\sum_{k=1}^{K}\left|{\hat{D}}_{eq,k}-D_{eq,k}\right|$$

The equivalent diameter was calculated as:

$$D_{eq}=2\sqrt{\frac{A}{\pi }}$$

where ${\hat{D}}_{eq,k}$ and $D_{eq,k}$ denote the equivalent diameters calculated from the predicted and ground-truth masks, respectively. A is the particle mask area after spatial calibration. Specifically, the calibrated area was calculated as:

$$A=N_{pix}s^{2}$$

where $N_{pix}$ is the number of pixels within the particle mask and s is the spatial calibration factor in μm/pixel.
